# Distribution of the CMV glycoprotein gH/gL/gO and gH/gL/pUL128/pUL130/pUL131A complex variants and associated clinical manifestations in infants infected congenitally or postnatally

**DOI:** 10.1038/s41598-019-52906-y

**Published:** 2019-11-08

**Authors:** Edyta Paradowska, Agnieszka Jabłońska, Mirosława Studzińska, Beata Kasztelewicz, Małgorzata Wiśniewska-Ligier, Katarzyna Dzierżanowska-Fangrat, Teresa Woźniakowska-Gęsicka, Justyna Czech-Kowalska

**Affiliations:** 1 Laboratory of Virology, Institute of Medical Biology, Polish Academy of Sciences, Lodz, Poland; 2 Department of Clinical Microbiology and Immunology, The Children’s Memorial Health Institute, Warsaw, Poland; 3 Department of Pediatrics, Immunology, and Nephrology, Polish Mother’s Memorial Hospital Research Institute, Lodz, Poland; 4 3rd Department of Pediatrics, Polish Mother’s Memorial Hospital Research Institute, Lodz, Poland; 5 Department of Neonatology and Neonatal Intensive Care, The Children’s Memorial Health Institute, Warsaw, Poland

**Keywords:** Herpes virus, Viral infection, Neurological manifestations

## Abstract

Human cytomegalovirus (CMV) is a major cause of morbidity in fetuses following intrauterine infection. The glycoprotein (g) envelope trimeric gH/gL/gO and pentameric gH/gL/pUL128/pUL130/pUL131A complexes are required for CMV entry into fibroblasts and endothelial/epithelial cells, respectively, and both are targets for neutralizing antibodies. The role of sequence variability among viral strains in the outcome of congenital CMV infection is controversial. Variation in the CMV *UL75* gene encoding glycoprotein H (gH), the *UL115* (gL), the *UL74* (gO), and the UL128 locus (UL128L) encoding three structural proteins (pUL128, pUL130, and pUL131A) was determined in 82 newborns with congenital CMV infection and 113 infants with postnatal or unproven congenital CMV infection. Genotyping was performed by sequencing analysis of PCR‐amplified fragments and the PCR-restriction fragment length polymorphism (RFLP) method, and the viral load was measured by quantitative real‐time PCR. The obtained results demonstrated that (1) different CMV variants and mixed CMV infections can be detected in newborns infected congenitally; (2) the gH1 genotype, *UL130* variant 6, and *UL131A* variant 1 were associated with some signs/symptoms within cohort of pediatric patients, mainly consisting of infants with symptomatic CMV infection. The results revealed that pUL130, pUL131A, and gH polymorphisms seemed to be associated with the outcome of CMV infection in infants.

## Introduction

Human cytomegalovirus (CMV) is an opportunistic ß-herpesvirus that infects 40% to nearly 100% of the adult population worldwide^1,2^. It is the most common cause of congenital infection worldwide and an important cause of morbidity and mortality in immunocompromised individuals. CMV infection is generally asymptomatic or mild in healthy individuals, whereas the virus undergoes latency in myeloid cells of the bone marrow and may reactivate at any time.

CMV is the leading viral cause of neonatal developmental disabilities, including sensorineural hearing loss (SNHL) and central nervous system (CNS) damage^3^. In the developed world, congenital CMV (cCMV) infections occur in 0.5–2.0% of pregnancies^4,5^. CMV infection can result in intrauterine growth restriction (IUGR), preterm birth, fetal or neonatal abnormalities and death. Approximately 5–10% of CMV-infected newborns are symptomatic, with findings including unilateral or bilateral SNHL, chorioretinitis, microcephaly, hepatomegaly, splenomegaly, thrombocytopenia, petechiae, jaundice, and seizures^4,6,7^. Moreover, approximately 15% of newborns with asymptomatic cCMV infection at the birth develop long-term neurological sequelae in the next years of life^8,9^. The highest risk of severe symptoms in the fetus and newborn exists in cases of primary maternal infection in the first-trimester^10^. However, natural immunity is not protective against CMV infection, and the majority of infected newborns are born to mothers who are already seropositive^7,11^. Primary or nonprimary maternal infection, including reactivation of the latent virus or reinfection with a different strain, can lead to crossing the placenta and infecting the fetus. Approximately 20–30% of nonimmune women who are infected during pregnancy transmit the virus to their offspring, whereas the rate of transmission following nonprimary infection is between 0.6–1%^7^.

CMV replicates in different cells, including epithelial cells, endothelial cells, fibroblasts and smooth muscle cells, which are the predominant targets^12^. This broad cell tropism facilitates both systemic spread within the host with efficient proliferation of the virus and interhost transmission. A number of factors can affect the interaction between the host immune system and the virus, and the major determinants of CMV disease appear to be host factors. The polymorphisms in the CMV genes encoding envelope glycoproteins, important targets of the host immune response, could potentially be associated with variability in the control of virus infection. Sequence variability among the viral strains may affect viral dissemination, disease severity, and immune response^13^. However, placental transmission appears to be independent of a specific virus strain, and different CMV genotypes are detected in infected neonates^14–19^. An association between CMV variants in surface proteins and clinical outcome is still controversial. The glycoprotein H (gH) is encoded by the *UL75* gene and has two genotype (gH1 and gH2) based on variability in the 37 amino acid N-terminal domain^13^. Both gH genomic variants do not correlate with symptomatic cCMV infection^20^; however, an association between the gH genotype and hearing loss in infants in another study was found^17^. The *UL74* gene encodes viral glycoprotein O (gO) and at least eight genetic variants of gO, including five major genotypes (gO1, gO2, gO3, gO4 and gO5) with sub-genotypes (gO1a, gO1b, gO1c, gO2a, gO2b), have been identified^21,22^. Genetic linkage between gO and glycoprotein N (gN), encoded by the *UL73* gene, has been reported in CMV-infected infants^22^, while strong genetic linkage between the gO1 and the gH1 genotypes has been found in immunosuppressed adult patients^23^. Nucleotide variations is high in gN and gO genes (40–50%), lower differences exist in glycoprotein B (gB) and gH genes (5–10%), while the glycoprotein L (gL) gene is highly conserved among clinical strains.

Herpesviruses use envelope glycoproteins to enter host cells, including the viral gB that is necessary for entry into all cell types^24^. This viral fusogen is highly immunogenic and is the target of neutralizing antibodies^25^. CMV gH is another dominant target of specific antibodies that can be strain-specific^26^. CMV gH is crosslinked through disulfide bonds with gL. CMV requires two membrane glycoproteins, gB and gH/gL, to enter host cells, but gH/gL binds cellular receptors before triggering gB^27^. It was also reported that gB and gH/gL form stable gB-gH/gL complexes in cell-free virions independent of receptor binding^28^. The gH/gL dimer exists on the CMV surface as part of a trimeric complex with gO (gH/gL/gO), known as the gCIII complex, or a pentameric complex with the UL128 protein (pUL128), pUL130 and pUL131A (gH/gL/pUL128-131A)^29^. gO and pUL128-131A bind to the same site on gH/gL through a disulfide bond with gL-Cys144. Introduction of a double mutation at the disulfide bond level of the pentamer impaired syncytium formation and reduced interference with CMV entry into epithelial cells^30^. The gH/gL/gO complex is made of three disulfide-bonded proteins, gH, gL, and gO and is sufficient for attachment to and infection of fibroblasts^31^. The platelet-derived growth factor receptor alpha (PDGFR-α) has been identified as a receptor for entry into cells^32–34^. It is suggested that the trimer complex may be required for entry into all cell types^33,35–37^. The gH/gL/gO trimer binds with high affinity through the gO subunit to PDGFR-α, which is expressed by fibroblasts but not by epithelial and endothelial cells^32,38^. It was recently shown that the N terminus of gO contributes to efficient spread in fibroblasts by promoting the interaction of virions with cellular PDGFR-α^39^. The gH/gL/pUL128-131A complex consists of five proteins, namely, gH, gL, pUL128, pUL130 and pUL131A; it is required for the infection of endothelial, epithelial, and myeloid cells but is dispensable for the infection of fibroblasts^40–45^. Pentamer-dependent entry into epithelial and endothelial cells by endocytosis followed by low-pH-dependent fusion, while CMV strains enter fibroblasts by pH-independent fusion with the plasma membrane^45^. The *UL128-131A* gene locus (UL128L) of CMV is indispensable for both productive infection of endothelial cells and viral transfer to leukocytes^31^.

Recent studies have revealed findings concerning pentamer structure, location of epitopes for neutralizing antibodies and potential binding sites for cell surface receptors^37^. These data suggest that receptor binding triggers a conformational change in the pentamer, allowing it to interact with gB and initiate the membrane fusion process. The dimer gH/gL is thought to act as an intermediary, transmitting the fusion trigger to gB^46,47^. It is suggested that complexes containing gH/gL play a key role in host cell tropism^32–34,48^. Moreover, high expression of the pentamer on the epithelial cell surface leads to the interference of virus entry into cells, possibly through sequestration of cell surface receptors, providing strong evidence for a cell-specific receptor^49^. High levels of pentamer expression have been associated with an increase in cell-association of the virus and with cell-to-cell transmission^50^. Pentamer proteins are the dominant target of the most potent neutralizing antibodies, highlighting their critical role in CMV infection^51,52^. While antibodies that target gB and gH/gL prevent infection of all cell types, antibodies specific to the pentamer are a thousand-fold more potent than antibodies against gB or gH/gL complex specifically in neutralizing CMV infection in epithelial and endothelial cells^32,52^. Moreover, antibodies against pentamer are capable of protecting cytotrophoblasts against CMV infection^53^. A response against pentamer in seronegative pregnant women with primary infection is associated with a lower risk of transmission of the virus to the fetus^54^. These findings make the gH/gL/pUL128-131A complex a promising vaccine candidate.

In this study, we determined the distribution of CMV genes that code proteins forming trimer and pentamer complexes in infants with congenital and postnatal infection. We show that some genomic variants are detected more frequently in congenital than in postnatal patients and are associated with an increased risk of specific disease outcomes. Because mainly symptomatic patients were enrolled in the study, our findings may not pertain to all CMV-infected infants. These studies confirm the findings that various CMV variants can be vertically transmitted. The results support the hypothesis that variability in pentamer genes is an important factor that affects clinical sequelae following CMV infection.

## Results

### Study population and clinical outcome

One hundred and ninety-five pediatric patients with CMV DNAemia were enrolled in the study. The patients were selected based on clinical diagnosis and diagnostic markers. Congenital CMV infection was confirmed in 82 newborns (positive CMV DNA in urine ≤ 21 day of life), and the remaining 113 children were classified as having postnatal or unproven congenital CMV infection (pCMV). Among infants in pCMV group, negative CMV DNA in urine ≤ 21 day of life was found in 83% cases. The average age at which the newborns with cCMV infection were examined was 9.6 days (median age 8.0 days; range 1–21 days), while in the group of infants with pCMV infection, it was 3.4 months (median age 3.0 months; range 1–11.5 months). The demographic and clinical characteristics of congenitally or postnatally infected children are summarized in Table 1. Almost all examined patients demonstrated cytomegaly symptoms, and this high frequency of symptomatic infection was due to selection bias. The most prevalent symptoms in CMV-infected children from both groups were neurological dysfunction and hematological disorders (especially anemia and thrombocytopenia). In addition, newborns with cCMV infection usually demonstrated CNS damage (73%), including microcephaly and abnormal brain ultrasound findings (cystic lesions, intracranial calcification, and ventriculomegaly). In contrast to postnatal infection, symptoms such as CNS damage (*P* < 0.0001), neurological dysfunction (*P* = 0.003), intrauterine growth restriction (*P* < 0.0001), unilateral or bilateral hearing loss (*P* < 0.0001), ocular defects (*P* = 0.015), thrombocytopenia (*P* = 0.0001), and petechiae (*P* < 0.0001) occurred more commonly after cCMV infection. Some complications, such as pneumonia and hepatitis were more common in infants in pCMV group (*P* = 0.005 and *P* = 0.022, respectively).

**Table 1:** Demographic and clinical characteristics of study subjects with CMV infection.

Characteristics	Type of infection		*P* ^a^
	cCMV	pCMV	
**Total No**.	82	113	
**Mean ± SD age**	9.6 ± 6.1 days	3.4 ± 2.2 months	
**Median (range) age**	8 (1–21) days	3 (1–11.5) months	
**Gender number; n (%)** ^**b**^			
Female	39 (47.6)	43 (38.1)	0.186
Male	43 (52.4)	70 (61.9)	
**Mean ± SD birth weight (g)**	2 763 ± 651	2 884 ± 874	0.06^c^
**Mean ± SD gestational age at birth (weeks)**	37.3 ± 4.6	37.1 ± 3.8	0.79^c^
**Symptoms/signs** ^**d**^ **; n (%)**	79 (96.3)	107 (94.7)	0.736
Neurological dysfunction^e^	60 (75.9)	58 (54.2)	**0.003**
CNS damage^f^	58 (73.4)	24 (22.4)	** < 0.0001**
-Cystic lesions	45 (57.0)	11 (10.3)	** < 0.0001**
-Microcephaly	21 (26.6)	3 (2.8)	** < 0.0001**
-Intracranial calcification	28 (35.4)	6 (5.6)	** < 0.0001**
-Ventriculomegaly	25 (31.6)	10 (9.3)	**0.0002**
IUGR	32 (40.5)	10 (9.3)	** < 0.0001**
Hearing loss	31 (39.2)	10 (9.3)	** < 0.0001**
Ocular defects	19 (24.1)	11 (10.3)	**0.015**
Liver damage	10 (12.7)	9 (8.4)	0.463
Hepatitis	6 (7.6)	21 (19.6)	**0.022**
Hepatomegaly	16 (20.3)	14 (13.1)	0.228
Splenomegaly	16 (20.3)	10 (9.3)	0.053
Hepatosplenomegaly	13 (16.5)	7 (6.5)	0.053
Pneumonia	3 (3.8)	19 (17.8)	**0.005**
Petechiae	16 (20.3)	2 (1.9)	** < 0.0001**
Jaundice	19 (24.1)	38 (35.5)	0.109
Hematological disorders^g^	51 (64.6)	72 (67.3)	0.755
-Anemia	42 (53.2)	60 (56.1)	0.766
-Thrombocytopenia	23 (29.1)	8 (7.5)	**0.0001**
**Anti-CMV serologic status; n (%)** ^**b**^			
IgG-positive, IgM-negative	46 (56.1)	62 (54.9)	1.000
IgG-positive, IgM-positive	32 (39.0)	42 (37.2)	0.891
IgG-negative, IgM-negative	0 (0)	6 (5.3)	0.083
No data	4 (4.9)	3 (2.6)	0.457

cCMV, congenital CMV infection; pCMV, postnatal or unproven congenital CMV infection; ^a^*P*, Fisher’s exact test; ^b^Values are the number of infants (%); ^c^*P*, Mann-Whitney *U* test with correction for continuity; ^d^Values are the number of infants with symptomatic CMV infection (%); ^e^Neurological dysfunction e.g. tremor, hypotonia/hypertonia, or poor sucking reflex; ^f^CNS damage; central nervous system damage e.g. cystic lesions, microcephaly, intracranial calcification, or ventriculomegaly; ^g^Hematological disorders e.g. anemia, thrombocytopenia, thrombocytosis, neutropenia, or leukocytosis; IUGR, intrauterine growth restriction.

### Viral load

The CMV DNA was detected in 94 samples obtained from 82 newborns with cCMV infection and in 125 samples obtained from 113 infants with postnatal or unproven congenital CMV infection. The viruria was found in all newborns with cCMV infection and in 83/113 (73.5%) of infants from pCMV group after 21 days of life. The CMV DNA concentration in blood samples obtained from congenitally infected newborns ranged from 2.1 × 10^2^ to 3.2 × 10^5^ copies/mL (median 4.2 × 10^3^ copies/mL; mean 3.8 × 10^4^ ± 8.6 × 10^4^ copies/mL), while in urine samples, it ranged from 3.1 × 10^2^ to 8.7 × 10^8^ copies/mL (median 1.2 × 10^6^ copies/mL; mean 3.4 × 10^7^ ± 1.2 × 10^8^ copies/mL). In infants from pCMV group, the viremia levels ranged from undetected to 8.2 × 10^5^ copies/mL (median 3.6 × 10^2^ copies/mL; mean 2.7 × 10^4^ ± 1.4 × 10^5^ copies/mL), whereas the viruria levels ranged from undetected to 2.2 × 10^8^ copies/mL (median 1.6 × 10^4^ copies/mL; mean 3.5 × 10^6^ ± 2.4 × 10^7^ copies/mL). In both patient groups, the CMV DNA concentration in urine was significantly higher than in blood samples (*P* < 0.0001, Wilcoxon test). The concentration of CMV DNA in urine samples was higher among the children with cCMV infection compared to the children who had pCMV infection (*P* < 0.0001; Mann–Whitney U-test). Similarly, the median blood virus load was significantly higher in children with cCMV infection than in those from pCMV group (*P* = 0.0002; Mann–Whitney U-test).

### Prevalence of CMV variants

**Figure 1:**
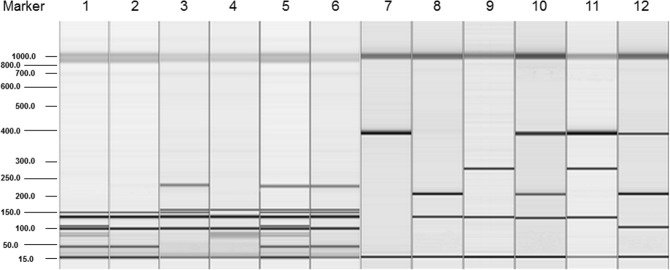
Visualization of PCR-RFLP products for gL and gO genotyping. Gel image: 1–6, gL variants; 7–12, gO variants. 1. gL1 (bands 40 bp, 90 bp, 100 bp, 108 bp, 133 bp, 150 bp), 2. gL2 (40 bp, 100 bp, 133 bp, 150 bp), 3. gL3 (100 bp, 133 bp, 150 bp, 165 bp, 225 bp), 4. gL4 (90 bp, 100 bp, 133 bp, 165 bp), 5. gL1 and gL3 (40 bp, 90 bp, 100 bp, 108 bp, 133 bp, 150 bp, 165 bp, 225 bp), 6. gL2 and gL3 (40 bp, 100 bp, 133 bp, 150 bp, 165 bp, 225 bp); 7. gO1 (390 bp), 8. gO2 (134 bp, 203 bp), 9. gO3 (134 bp, 271 bp), 10. gO1 and gO2 (134 bp, 203 bp, 390 bp), 11. gO1 and gO3 (134 bp, 271 bp, 390 bp), 12. gO1 and gO4 (110 bp, 203 bp, 390 bp). Alignments markers (15 bp, 1 kbp).

The trimer gH/gL/gO gene products were amplified for all examined pediatric patients (Table 2). Genotyping of gH was performed by analysis of nested PCR (nPCR)-amplified fragments and the two genomic variants were identified by different amplicon size lengths. The applied PCR-restriction fragment length polymorphism (PCR-RFLP) method allowed the detection of major gL or gO genotypes (Fig. 1). Genotypes determined by RFLP were sequenced, and no discrepancies were found. In contrast, the UL128 locus gene products were amplified and sequenced successfully for 79/82 (96.3%) newborns with cCMV infection and 101/113 (89.4%) infants in pCMV group (Table 3).

**Table 2:** Distribution of CMV gH/gL/gO genotypes in infants infected congenitally (n = 82) or postnatally (n = 113).

Gene	Genotype	Prevalence of CMV genotypes, n (%)		*P* ^a^
		cCMV	pCMV	
*UL74*	gO1	75 (91.5)	103 (91.2)	1.000
	gO2	7 (8.5)	4 (3.5)	0.207
	gO3	5 (6.1)	6 (5.3)	1.000
	gO4	20 (24.4)	12 (10.6)	**0.018**
	mixed	26 (31.7)	13 (11.5)	**0.0009**
*UL75*	gH1	54 (65.9)	63 (55.8)	0.183
	gH2	46 (56.1)	74 (65.5)	0.233
	mixed	18 (22.0)	24 (21.2)	1.000
*UL115*	gL1	40 (48.8)	46 (40.7)	0.307
	gL2	23 (26.8)	36 (31.8)	0.637
	gL3	50 (61.0)	31 (27.4)	**<0.0001**
	gL4	23 (28.0)	37 (32.7)	0.532
	mixed	46 (56.1)	34 (30.1)	**0.0004**
*UL74-UL75-UL115*	mixed	62 (75.6)	55 (48.7)	**0.0002**

n, number of isolates with CMV genotype; cCMV, congenital CMV infection; pCMV, postnatal or unproven congenital CMV infection; ^a^*P*, Fisher’s exact test.

**Table 3:** Distribution of CMV UL128L (UL128, UL130, and UL131A) variants in infants infected congenitally or postnatally.

Gene	Variant	Prevalence of CMV variants, n (%)		*P* ^a^
		cCMV	pCMV	
***UL128***	1	10 (12.7)	6 (5.9)	0.186
	2	2 (2.5)	8 (7.9)	0.189
	3	34 (43.0)	45 (44.6)	0.880
	4	7 (8.9)	5 (4.9)	0.372
	5	13 (16.5)	16 (15.8)	1.000
	6	7 (8.9)	18 (17.8)	0.127
	7	3 (3.8)	2 (1.9)	0.655
	8	1 (1.3)	2 (1.9)	1.000
	9/10	1 (1.3)	1 (0.9)	1.000
	11	3 (3.8)	1 (0.9)	0.321
	12	0	1 (0.9)	1.000
	13	1 (1.3)	2 (1.9)	1.000
	Mixed	3 (3.8)	7 (6.9)	0.516
***UL130***	1	6 (7.6)	13 (12.9)	0.331
	2	2 (2.5)	2 (1.9)	1.000
	3	3 (3.8)	3 (2.9)	1.000
	4	2 (2.5)	9 (8.9)	0.116
	5	1 (1.3)	7 (6.9)	0.081
	6	37 (46.8)	44 (43.6)	0.763
	7	6 (7.6)	13 (12.9)	0.331
	8	11 (13.9)	4 (3.9)	**0.027**
	9/10	5 (6.3)	1 (0.9)	0.088
	11	0	1 (0.9)	1.000
	12	0	2 (1.9)	0.505
	13	2 (2.5)	0	0.191
	14/15/16	1 (1.3)	0	0.439
	17	2 (2.5)	1 (0.9)	0.583
	18	1 (1.3)	2 (1.9)	1.000
	19	2 (2.5)	0	0.191
	Mixed	8 (10.1)	2 (1.9)	**0.023**
***UL131A***	1	63 (79.7)	59 (58.4)	**0.002**
	2	0	3 (2.9)	0.257
	3	0	1 (0.9)	1.000
	4	16 (20.3)	35 (34.7)	**0.045**
	5	0	2 (1.9)	0.505
	6	1 (1.3)	0	0.439
	7	1 (1.3)	2 (1.9)	1.000
	Mixed	2 (2.5)	1 (0.9)	0.583

n, number of infants with CMV variant; cCMV, congenital CMV infection; pCMV, postnatal or unproven congenital CMV infection; ^a^*P*, Fisher’s exact test.

The distribution of the gH genotypes were similar in both patient groups (*P* > 0.05). To explore the data, one hundred and five infants with CMV infection, including 28 cases of cCMV and 77 cases of pCMV, were examined for the presence of *UL75* genotypes as described in our previous study^17^. No significant advantages of the gH1 genotype in congenital cases and gH2 in postnatal cases were observed. The gH1 genotype was detected in 54/82 (65.9%), while the gH2 in 46/82 (56.1%) newborns with cCMV infection (Table 2). Among children with pCMV infection, the gH1 genotype was found in 63/113 (55.8%), while the gH2 genotype was found in 74/113 (65.5%) cases. The nucleotide sequence analysis confirmed the gH genomic variants previously identified by a nPCR analysis. Mixed infections with both gH1 and gH2 genotypes were detected in 18/82 (22.0%) patients with cCMV infection and in 24/113 (21.2%) cases with pCMV infection.

**Figure 2:**
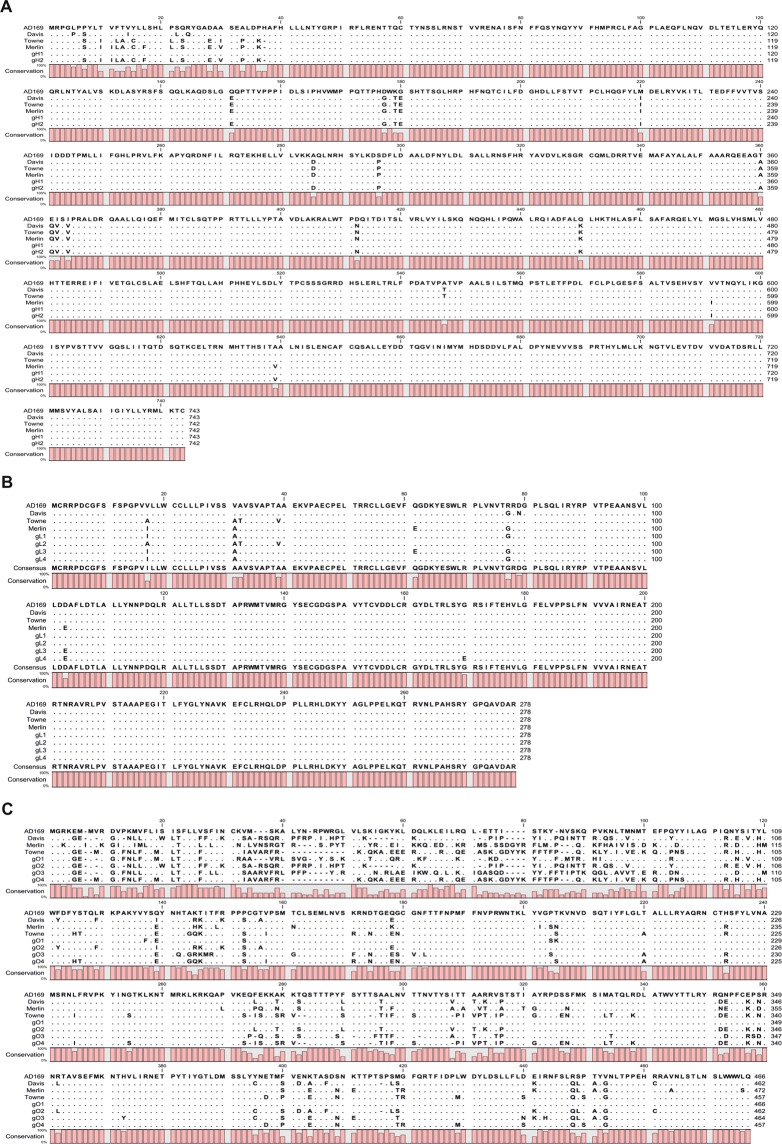
Amino acid alignments of gH (**A**), gL (**B**), and gO (**C**) in clinical isolates from children with congenital and postnatal CMV infection.

The *UL115* gene encoding gL had a low proportion of nucleotide and amino acid variability in clinical isolates (5–9% and 1.4–2.5%, respectively), while low sequence conservation for *UL74* (gO) was observed (sequence variability: 20–50% and 19.4–26.4%, respectively). All obtained amino acid sequences are illustrated in Fig. 2A–C. The gL3 genotype was prevalent in congenital infections, whereas this genomic variant was less common in postnatal infection (*P* < 0.0001). The most prevalent gO genotype in both groups, newborns with cCMV infection and infants with pCMV infection, was gO1 (approximately 91%). The gO4 genotype was detected more frequently in congenital compared to postnatal infection (*P* = 0.018). Congenital infection with mixed gL and gO genotypes was detected in 56.1% and 31.7% cases, respectively. Mixed pCMV infections were detected in 30.1% (gL) and 11.5% (gO) of infants. The children exhibited mixed infection with two gO genotypes, especially gO1 and gO4 or gO3, and with two or three distinct CMV gL genotypes. Among examined infants, mixed infections were identified mostly with viral gL1-gL3 (29 cases), gL2-gL3 (22 cases), gL3-gL4 (14 cases), and gL1-gL3-gL4 (5 cases) genomic variants. Analysis of the gH/gL/gO genotypes revealed that multiple CMV strains were detected in congenitally infected patients. Unexpectedly, considering all trimer gH/gL/gO genotypes, mixed infections were more commonly detected in congenital than in postnatal infections (75.6% *vs*. 48.7%; *P* = 0.0002).

**Figure 3:**
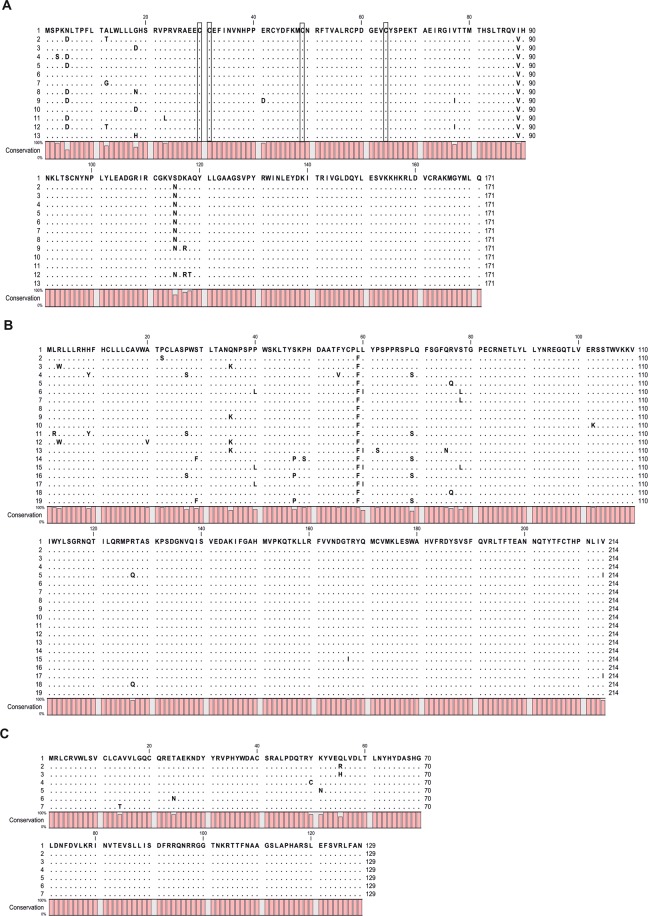
The phylogenetic analysis of UL128 (**A**), UL130 (**B**), and UL131A (**C**) from clinical isolates of newborns with congenital CMV infection and infants with postnatal or unproven congenital CMV infection. The conserved cysteine residues are in box.

The obtained *UL128*, *UL130*, and *UL131A* gene sequences were clustered into thirteen (named from 1 to 13), nineteen (1–19), and seven (1–7) variants, respectively. An amino acid sequence alignment of the UL128, UL130, and UL131A products is shown in Fig. 3A–C. The UL128L showed a low degree of variability. The ratios of sequence variability of UL128 ranged from 3 to 9% at the DNA level and from 0.6 to 2.3% at the amino acid level. The overall variability of the UL130 ranged from 2 to 10% at the DNA level and from 0.5 to 2.8% at the amino acid level, whereas the variability of the UL131A was 2–4% at the DNA level and 0.8% at the amino acid level.

Several viral variants were identified in newborns with cCMV infection, indicating that all variants of the virus could be passed from mother to child. The *UL128* variant 3 was identified most commonly in both pediatric patient groups (43.0% and 44.6%), and no statistically significant association with the *UL128* variant distribution among children with congenital or postnatal CMV infection was observed (Table 3). The *UL130* variant 6 was prevalent in newborns with cCMV and infants in pCMV group (46.8% and 43.6%, respectively). The *UL130* variant 8 was identified more commonly in cCMV than pCMV patients (*P* = 0.027). Analysis of the *UL131A* region in clinical isolates revealed that the variant 1 was prevalent in both patient groups, and it occurred more frequently in congenital than postnatal infections (79.7% *vs*. 58.4%; *P* = 0.002). In contrast, the *UL131A* variant 4 was found in 20.3% newborns with cCMV and in 34.7% infants in pCMV group (*P* = 0.045). Some mixing of pUL128, pUL130, and pUL131A gene variants was observed, but the frequency was ≤ 10% among isolates.

### Linkages among components of the gH/gL/gO complex

Potential linkage disequilibrium between the gH, gO, and gL genotypes was investigated in isolates obtained from all pediatric patients. The analysis included only those isolates for which the genotypes were determined (Table 4). One hundred and thirteen of 122 isolates with the gH1 genotype had also a gO1 genotype (92.6%), 2 had gO2 (1.6%), 1 had gO3 (0.8%), and 6 had gO4 (4.9%). Among 123 isolates with gH2 genotype, 112 had also a gO1 genotype (91.1%). Mixed CMV infections with various gO genotypes were detected in 25/122 (20.5%) isolates with gH1 genotype and in 18/123 (14.6%) isolates with gH2 genotype. The prevalent gO1 genotype was not associated with gH genomic variant (*P* > 0.05). Analysis of the Spearman’s rank correlation coefficients (ρ) and associated *P*-values showed no significant correlation between the gH, gL, and gO genotypes, including gH-gO (ρ = 0.02), gH-gL, and gL-gO (ρ = 0.12) paired groups (Spearman’s correlation test *P* > 0.05). In addition, there was no significant association between the gH, gO, and gL genotypes (Cohen’s kappa coefficient ĸ = −0.07; *P* > 0.05). We observed a weak negative correlation between pUL130 and pUL131A variants (ρ = −0.18; *P* < 0.05). No significant association was found between other gene pairs encoding the gH/gL/pUL128-131A complex (ρ-values from −0.05 to 0.11; *P* > 0.05). In the examined patients we also observed no conformity between the gH, gL, pUL128, pUL130, and pUL131A variants (ĸ = −0.05; *P* > 0.05).

**Table 4:** Distribution of genotypes of the gH, gL, and gO in isolates from children with CMV infection.

gL genotype	No. of isolates with gH1 and gO genotype					No. of isolates with gH2 and gO genotype				
	1	2	3	4	mixed	1	2	3	4	mixed
1	21	0	1	2	6	17	3	0	1	4
2	15	1	0	0	4	16	2	0	1	2
3	7	0	0	0	1	7	0	0	0	1
4	16	1	0	1	3	14	0	1	0	4
mixed	29	0	0	3	11	41	0	1	1	7

### CMV gene variability is associated with an increased risk of specific disease outcomes

In the patients carrying the gH1 genotype, hearing loss was diagnosed with higher incidence compared to those with the gH2 genotype (*P* = 0.023; Table 5). As was previously found in a smaller group of cases^17^, infants carrying the gH2 genotype were diagnosed with deafness at a lower incidence compared to those with the gH1 genotype (*P* = 0.004). In addition, gH2-infected children exhibited an increased risk of developing purpuric and petechial rashes. The gH2 genotype was associated with a diminished risk of hearing loss and psychomotor retardation in infants in adjusted and unadjusted models (Table 5).

**Table 5:** CMV variants as prognostic markers for the risk of symptoms in infants with cCMV or pCMV infection.

Gene	Genotype/variant	Symptom/sign	n (%)	Unadjusted		Adjusted	
				*P*	OR (95% CI)	*P* ^a^	OR (95% CI)
		**cCMV**					
***UL75***	gH1	Neurological dysfunction^b^	44/60 (73.3)	0.021	3.3 (1.2–9.1)	0.027	3.3 (1.1–9.5)
	gH2	Petechiae	13/16 (81.3)	0.033	4.3 (1.1–16.6)	0.023	6.5 (1.3–33.0)
***UL115***	gL2	Hematological disorders^c^	14/51 (27.5)	0.025	0.1 (0.0–0.7)	0.032	0.1 (0.0–0.8)
***UL130***	6	IUGR	20/32 (62.5)	0.013	3.2 (1.3–8.2)	0.006	3.9 (1.5–10.2)
	6	Ocular defects	13/19 (68.4)	0.024	3.5 (1.2–10.5)	0.021	3.8 (1.2–11.7)
***UL131A***	1	Neurological dysfunction	29/60 (48.3)	0.025	4.5 (1.2–17.2)	0.026	4.6 (1.2–17.8)
	1	Hematological disorders	35/51 (68.6)	0.032	0.2 (0.1–0.9)	0.034	0.2 (0.1–1.1)
		**pCMV**					
***UL131A***	1	Hepatitis	16/21 (76.2)	0.021	3.6 (1.2–10.1)	0.013	4.0 (1.3–11.9)
		**All cases**					
***UL75***	gH1	Hearing loss	31/41 (75.6)	0.023	2.5 (1.1–5.4)	0.027	2.6 (1.1–5.9)
	gH1	Microcephaly	19/24 (79.2)	0.046	2.9 (1.0–8.0)	0.083	2.7 (0.9–8.1)
	gH2	Petechiae	19/24 (79.2)	0.056	3.5 (1.0–12.4)	0.019	6.1 (1.4–27.8)
	gH2	Psychomotor retardation	37/74 (50.0)	0.011	0.5 (0.3–0.8)	0.021	0.5 (0.3–0.9)
	gH2	Hearing loss	17/41 (41.5)	0.004	0.4 (0.2–0.7)	0.018	0.4 (0.2–0.9)
***UL115***	gL2	Hematological disorders	36/124 (29.0)	0.018	0.2 (0.0–0.7)	0.013	0.2 (0.0–0.7)
***UL130***	6	IUGR	25/42 (59.5)	0.008	2.6 (1.3–5.2)	0.004	3.0 (1.4–6.2)
***UL131A***	1	IUGR	36/42 (85.7)	0.001	4.6 (1.8–11.6)	0.003	4.3 (1.6–11.0)
	1	CNS damage^d^	60/82 (73.2)	0.005	2.4 (1.3–4.5)	0.015	2.3 (1.2–4.7)

n, number of genotype/variant cases among symptomatic patients; OR, odds ratio; 95% CI, 95% confidence interval; cCMV, congenital CMV infection; pCMV, postnatal or unproven congenital CMV infection; ^a^Adjusted analysis was carried out for CMV DNA copy number in urine samples; ^b^Neurological dysfunction e.g. tremor, hypotonia/hypertonia, or poor sucking reflex; ^c^Hematological disorders e.g. anemia, thrombocytopenia, thrombocytosis, neutropenia, or leukocytosis; ^d^CNS damage e.g. cystic lesions, microcephaly, intracranial calcification, or ventriculomegaly; IUGR, intrauterine growth restriction.

Infection with gH1 genotype was also associated with a three-fold increased risk of neurological dysfunction and microcephaly (*P* < 0.05). Infection with the gH2 genotype was associated with a decreased risk of hearing loss and ventriculomegaly (*P* = 0.007 and *P* = 0.024, respectively), though this genotype was observed only in a small number of the symptomatic infants (data not shown).

The *UL130* variant 6 was detected in 62.5% of newborns with IUGR (*P* = 0.013) and was associated with at least a three-fold increased risk of this symptom (Table 5). The risk of ocular defects was more than three-fold increased following congenital infection with this variant (*P* = 0.024). Infection with the *UL131A* variant 1 was associated with a four-fold increased risk of IUGR, neurological dysfunction, and hepatitis (*P* = 0.001, *P* = 0.025, and *P* = 0.021, respectively) and with at least a two-fold increased risk of CNS damage (*P* = 0.005). In contrast, gL2-infected patients exhibited a decreased risk of developing hematological disorders (*P* = 0.025 for neonates with cCMV infection; *P* = 0.018 for all pediatric patients).

### CMV glycoprotein polymorphisms and viral load

The viral load in the blood and urine samples correlate with the infection of some CMV genotypes. The gL2 and gO3 genotypes were associated with a low shedding of CMV in urine (*P* = 0.005 and *P* = 0.008, respectively; Wilcoxon test). The viral load in the blood and urine samples did not correlate with the presence of the other CMV variants.

## Discussion

Several studies have analyzed sequences from CMV clinical isolates and revealed that genomic variability caused additional infectivity or immunomodulation functions^55–60^. The increasing evidence suggests a correlation of clinical importance of CMV genetic diversity with pathogenesis^15–18,20,61^. To assess whether the variability in the CMV genes encoding trimeric and pentameric complexes are associated with congenital transmission, we compared the frequencies of variants in children with and without confirmed congenital infection. Our study focused on the variation in the all CMV genes encoding envelope protein complexes, gH/gL/gO and gH/gL/pUL128-131A, and the distribution of genotypes in pediatric patients. We have determined the sequence variation in the *UL128-UL131A* genes, named these specific viral variants and identified variants transmitted during congenital and postnatal infection. Our study provides the first demonstration that the CMV variants of the pentamer complex seems to be assigned to a clinical outcome in children, including, gH1, *UL130* variant 6, and *UL131A* variant 1. In addition, the gL3 and gO4 genotypes of CMV might be an important virological marker of congenital infection in children.

Detailed sequence analysis showed that *UL128*, *UL130*, and *UL131A* genes were highly conserved in clinical isolates, suggesting an important role of these genes in viral pathogenesis^55,61–63^. Most amino acid substitutions were limited to the N-terminal region of the *UL128* and *UL131A* genes, although they can also be found in other parts of the *UL130* gene. The UL128 and UL130 proteins share conserved cysteine residues that are characteristic of CC- and CXC-chemokines, respectively. Our findings showed 96–99%, 84–97%, and 97–99% sequence identity at the aa level in the UL128, UL130, and UL131A proteins, respectively, in comparison to the protein sequences obtained from the publishing database NCBI. The overall identity of these CMV genes was similar and ranged between 92% and 96% in isolates from Italian patients^55^. Sun *et al*. analyzed the UL128L in 23 congenitally infected Chinese infants and found that the nucleotide and amino acid identity among strains scored 96–97%^63^. Baldanti *et al*. suggested that a high conservation of the UL128L is essential for CMV growth in endothelial cells and virus transfer to leukocytes^55^. According to the results of Vogel *et al*. sequencing with subsequent phylogenetic analysis showed that all UL128L genes of all clinical isolates from AIDS patients were highly conserved^61^. The conserved cysteine residues in exons 2 and 3 of UL128 were detected in all variants^61^. These conserved cysteines in CMV UL128 protein are especially important for CC chemokine motifs. Vogel *et al*. found no evidence for a connection between UL144/UL128/UL130/UL131A genotypes and the incidence of CMV retinitis in AIDS patients^61^. We have observed that none of the detected *UL128* variants were significantly linked with symptomatic CMV infection. Our results revealed that the *UL130* variant 6 and the *UL131A* variant 1 were associated with an increased risk of specific symptoms in children. No significant difference was detected between the CMV variants determined on the basis of the UL128L and the signs of infection in Chinese infants^63^. The *UL130* variant 6 showed a few amino acid substitutions such as P40L, L60I, and S78L that were undetected in common CMV variants. It was found that a 2-bp insertion (TT) led to a frameshift mutation in the *UL130* (Towne strain) and a 1-bp insertion (A) in *UL131A* (AD-169 strain) genes leading to a frameshift mutation have impaired the immunogenicity of viral strains^58,59^. The mutations include nucleotide substitutions that introduce in-frame translational termination codons or splicing are predicted to ablate gene functions, insertions, and deletions that affect one or more genes. These mutations impact the growth properties and tropism of the virus. Moreover, these changes have likely impaired the immunogenicity, particularly with respect to induction of epithelial cell and endothelial cell neutralizing antibodies^57,60^.

The findings of Maidji *et al*. support a potential role of endothelial cells in CMV transmission from the uterus to endovascular cytotrophoblasts^64^. The syncytiotrophoblast constitutes a barrier to vertical transmission, and first-trimester chorionic villi are largely resistant to CMV infection, whereas cytotrophoblasts and other villous cells are susceptible^65^. It is suggested that virions transmitted to cytotrophoblasts could then spread the infection to the placenta and to fetal blood vessels in the villus core. Considering the structure of the human placenta as an active barrier against infection, the pentamer gH/gL/pUL128-131A complex is of utmost importance in vertical CMV transmission^53^. The pUL128/pUL130/pUL131A, when assembled with the gH/gL heterodimer to form the pentameric complex, is necessary for entry into endothelial and epithelial cells, as well as for CMV transmission to leukocytes. The pentamer complex mediates virus entry into epithelial and endothelial cells by endocytosis and fusion. The entry process into fibroblasts is independent of the proteins of the pentamer complex, and the UL128L genes may be lost during long-term cultivation in human fibroblasts. Thus, this genetic locus is supposed to play an important role in cell tropism. Recent studies of the virus tropism concentrated on the UL128L of wild-type CMV strains. The pUL128/pUL130/pUL131A complex is also the target of the most potent neutralizing antibodies following natural infection^31,41,52^. The neutralization potency of CMV infection in epithelial cells by monoclonal antibodies against the UL128L complex and the gH/gL complex was 1000-fold and 10-fold higher, respectively, than that by CMV-specific hyperimmune globulin^66^. The pentamer gH/gL/pUL128-131A complex is of interest in the CMV vaccine field.

gH/gL dimer may be complexed either with pUL128L forming the pentamer complex, or gO, forming the gH/gL/gO complex. Greater amounts of pentamer gH/gL/pUL128/pUL130/pUL131A complex may result in there being less gH/gL available for the formation of trimer gH/gL/gO complex. Two viral factors, UL148 and US16, were identified to impact the composition of gH/gL complexes in CMV strains^67,68^. Moreover, the expression levels of UL128-131A and gO seem to influence the abundance of gH/gL/pUL128-131A and gH/gL/gO complexes and virion infectivity^69^. There are at least eight genotypes of gO that differ by 10 to 30% of amino acids. Used set of primers was adequate for amplifying a region of the *UL74* gene that was digested for identification of the five major gO genotypes, with two of them divided in five sub-genotypes. However, no relationship was observed between gO genotype and the outcome of CMV infection in infants. The distribution of the gO genotypes in clinical isolates from different disease settings confirmed that no correlation exists between gO type and CMV disease^23^. The present results performed on larger groups of pediatric patients confirmed our earlier findings that infection with the gH2 genotype diminishes the risk of hearing loss, whereas detection of the gH1 genotype is associated with hearing loss^17^. In addition, an association between the gH1 genotype and the incidence of neurological dysfunction was found. The heterogeneity between gH1 and gH2 genotypes is characterized by the deletion of a proline at position 36 and the substitution of lysine for histidine at position 37 in the AD-169 strain.

Most of congenital infections occur during nonprimary maternal infection, and it has been estimated that approximately three-quarters of congenital CMV infections occur in the setting of recurrent maternal infection during pregnancy^5^. A previously acquired maternal CMV infection does not provide complete protection against infection of the fetus, but it reduces this risk. It was found that antibodies targeting the CMV pentameric complex are able to neutralize genetically diverse clinical isolates of CMV^66^. Moreover, a pentameric complex of proteins elicited far more neutralizing antibodies than gB^41^. Antibodies to anti-UL128L complex are increased in the groups of non-transmitting mothers^54^. The increase in antibodies after CMV infection in pregnant women is associated with a decreased risk of congenital infection, suggesting that anti-UL128L complex antibodies play a critical role in protection against transmission to the fetus^54,70^. However, it is unknown if antibodies specific for pentamer complex can block infection of trophoblasts and restrict cCMV transmission. It was observed that these antibodies inhibit infection of term cytotrophoblasts, while no inhibition in the first-trimester trophoblast progenitor cells was found^53,71^.

This study has a number of strengths, but also some limitations. This is the first study to focus on the genes encoding the gH/gL/pUL128-131A complex of CMV in infants infected congenitally or postnatally. The main strength of the present study is the clinical evaluation of children with a CMV infection and important clinical implications. We have found significant associations of CMV variants with specific clinical symptoms. It should be noted that our results are not representative of the entire infant population and that the detected CMV variant distribution is only valid for symptomatic patients. Because the sample population of patients with specific symptoms was small, the significance of these results must be handled with caution. Further studies using larger patient groups from different geographical regions are needed to confirm our findings. Moreover, investigations of the role of intra-host recombination and functions of variants of key viral genes are necessary to elucidate the role of CMV diversity in pathogenesis. It should be underlined that we have no universal CMV screening for newborns in our country. As pCMV infection was diagnosed in children above three weeks of age, it is possible that, apart from the postnatally infected majority, there are also congenitally infected infants (with no clinical findings at birth) in this group.

In summary, this report demonstrates that a relationship between the CMV variant in trimeric and pentameric complexes, including gH, pUL130 and pUL131A, and clinical outcome exists. This study suggests that the CMV variant may be one of the virological markers of congenital CMV disease.

## Methods

### Patients

A total of 195 children with CMV infection were enrolled from February 2008 to April 2011 and again from January 2015 to August 2018 at the Children’s Memorial Health Institute, Warsaw, and the Polish Mother’s Memorial Hospital Research Institute in Lodz, Poland. The geographical origins of the patients were in half the Central Poland (Warsaw, Lodz and the surrounding areas) and all Poland. Patients were divided into two groups: cCMV group, which represent infants who were CMV-positive in urine samples collected ≤ 21st day of life, and pCMV group, which represented infants with the postnatal acquisition of CMV (CMV-negative in urine samples ≤ 21st day of life, seroconversion > 21 day of life) or probable postnatal CMV infection/unproven cCMV infection (unavailable result of CMV DNA in the urine ≤ 21st day of life). Blood and/or urine samples were obtained from 82 newborns with cCMV infection and from 113 infants in pCMV group. One hundred and five children with CMV infection, including 28 cases of cCMV and 77 cases of pCMV, were earlier examined for the presence of *UL75*, as described in our previous study^17^. CMV infection was confirmed by the presence of CMV-specific antibodies and/or CMV DNA detection in whole blood and/or urine samples. Congenital infection was confirmed within the first 2–3 weeks of life by CMV DNA detection in the urine samples. When the samples were collected after 3 weeks of life, infants were classified as having postnatal or unproven congenital CMV infection. The children were classified as having symptomatic infection with any clinical manifestations (jaundice, petechiae, IUGR, hepatosplenomegaly, hepatitis, cholestasis, hearing loss, microcephaly, neurological dysfunction, CNS damage in neuroimaging [ultrasound and/or magnetic resonance imaging], chorioretinitis, pneumonia) or laboratory findings, including thrombocytopenia, granulocytopenia, and anemia. Due to selection bias, 96.3% of newborns and 94.7% of infants with CMV infection in this study were symptomatic. Serum samples were assessed for anti-CMV IgG and IgM antibodies with the use of ELISA AxSYM CMV IgM and IgG (Abbott Laboratories, Abbott Park, IL, USA) or CLIA LIASON CMV IgM and IgG assays (DiaSorin, Saluggia, Italy). The demographic and clinical characteristics of pediatric patients with CMV infection were summarized in Table 1. The study protocols were approved by the Bioethics Committee of the Medical University of Lodz (RNN/278-279/16/KE, RNN/120/09/KE) and the Ethics Committee of the Polish Mother’s Memorial Hospital Research Institute (90/KBE/207). Parents or guardians provided written informed consent to participate in this study on behalf of the children. All experiments were performed in accordance with relevant guidelines and regulations.

### DNA extraction

Total genomic DNA was extracted from peripheral blood and urine samples using a QIAamp DNA Blood Mini Kit or QIAamp DNA Mini Kit (Qiagen GmbH, Hilden, Germany) according to the manufacturer’s instructions. The concentration and purity of DNA were assessed using a 2000c UV-VIS Spectrophotometer (Thermo Scientific, Wilmington, DE, USA).

### Quantification of CMV DNA

The CMV DNA copy numbers in DNA isolates were determined using a 7900HT Fast Real-Time PCR system (Applied Biosystems, Foster City, CA, USA) as previously described^72,73^. DNA was amplified using primers specific for the CMV gB (*UL55*) gene. The sensitivity of the assay was determined to be approximately 2 × 10^2^ copies/mL. A negative control without template DNA was included in every amplification run.

### Genotyping of three genes encoding the gH/gL/gO complex of CMV

The *UL75* gene (gH) was amplified by nPCR by using two sets of primers, as described elsewhere^17^. The genomic variants gH1 and gH2 were identified by different amplicon size lengths (240 bp and 237 bp, respectively). The identification of *UL74* (gL) and *UL115* (gO) was performed by nPCR-RFLP with the primers 5′-TAACGGGCGCTTGTTTACGT-3′ and 5′-CAGCAAAACGACCAGAATCAG-3′ (*UL74*) and 5′-GACGCACGGCGCGGTTGGTACG-3′ and 5′-CGTGCCGCAGACTTGATGTGCCG-3′ (*UL115*) in the first round and primer sets designed in our Laboratory or described previously by Stanton *et al*.^74^. The sequences of all primers used in PCR assays are listed in Table 6. Each run consisted of an initial denaturation at 95 °C for 1 min following by 45 consecutive cycles of denaturation at 95 °C for 3 min and different annealing temperatures (68 °C for *UL74* and 55 °C for *UL115*) for 30 s, extension at 72 °C for 1 min, and the final extension at 72 °C for 10 min. The PCR was performed in a Veriti® 96 Well Thermal Cycler (Applied Biosystems). Each PCR was performed in a volume of 50 μl as follows: 0.5 μg (blood) or 0.2 μg (urine) template DNA (5 μl), 5 μl 10 × DreamTaq™ Buffer (20 mM Tris-HCl, pH 8.0; 1 mM DTT, 0.1 mM EDTA, 100 mM KCl, 0.5% Nonidet P40, 0.5% Tween 20, 50% glycerol), 4 μl 2.5 mM dNTP, 0.5 μl gene-specific primers (100 pmol/μl of each), 0.25 μl DreamTaq™ polymerase (5 U/μl, Fermentas, Glen Burnie, MD, USA), and 34.75 μl nuclease-free water. DNA isolates from MRC-5 cells (ATCC CCL-171, American Type Culture Collection, Rockville, MD, USA) infected with the CMV strains AD-169 (ATCC VR-538), Towne (ATCC VR-977) or Davis (ATCC VR-807) was used as positive controls in each of PCR runs, and nuclease-free water was used as a negative control. The nPCR products were digested with the restriction enzymes HpaII (*UL74*), Eco24I [BanII], and CseI [HgaI] (*UL115*) (Fermentas, Hanover, MD, USA) according to the manufacturer’s recommendations. The restriction enzymes and the CMV genotypes identified by different fragment lengths are described in Table 7. Briefly, a digestion with *Hpa*II was used to characterize the five major gO genotypes, while double digestion with *Eco24*I and *Cse*I was used to identify the four gL genotypes. The nPCR products and the digested DNA were analyzed using a QIAxcel DNA Screening Kit and a QIAxcel system (Qiagen) and the AL420 method. The QX Alignment Marker 15 bp/1 kbp and QX DNA Size Marker 50–800 bp were included in the analysis. Randomly selected samples (50–60 samples per each gene) were sequenced using the BigDye Terminator v3.1 Cycle Sequencing Kit and the 96-capillary 3730xl DNA Analyzer (Applied Biosystems).

**Table 6:** Primer sequences and amplicon lengths obtained in nested PCR assays in the CMV genes.

Gene	Starter	Primer sequences 5’ → 3’	Amplicon length (bp)	References
***UL74***	F	TAACGGGCGCTTGTTTACGT	868	This paper^74^
	R	CAGCAAAACGACCAGAATCAG		
	nF	TAGATTCCGGCTCATGGCGTT	356	This paper
	nR	CCCAGCTTAGAAAACCCGCAA		
***UL75***	F	TGTTACGGAGGCTGCTGTTG	334	^17^
	R	GACGCGACTTTTGTAACCCG		
	nF	TCCTGGGATCCTTTCTCTCCTTCT	235	^75^
	nR	ATGGGTCTCCCGTAGGTGTTG		
***UL115***	F	GACGCACGGCGCGGTTGGTACG	636	This paper
	R	CGTGCCGCAGACTTGATGTGCCG		
	nF	CGGTGGCACCAGCTCGAAGCCT	558	This paper
	nR	ATGTGCCGCCGCCCGGATT		
***UL128***	F	TCGGCGATAAACACCACTATC	910	^61^
	R	CCATCCCAATCTCATCGTTT		
	nF	GCGTATTTCGGACAAACACACA	850	^61, 76^
	nR	CCATCCCAATCTCATCGTTT		
***UL130***	F	TCGGCGATAAACACCACTATC	2182	^61^
	R	GCTCAGAGATCCCGAGTACG		
	nF	GCGGTTTGGAATACGTCA	478	This paper
	nR	ACCGAGGCCAATAACCAGAC		
***UL131A***	F	TCGGCGATAAACACCACTATC	2182	^59^
	R	GCTCAGAGATCCCGAGTACG		
	nF	TGAAAGTGGTGACGAAGCAG	569	^59^
	nR	TCTTTCTCAGTCTGCAACATGCG		^76^

bp, base pairs; F, forward starter; R, reverse starter; nF, nested-forward starter; nR, nested-reverse starter.

**Table 7:** Restriction enzymes and length of the restriction fragments.

Gene	Restriction enzyme	Genotype	Length of the restriction fragments (bp)
***UL74***	*HpaII*	gO1	390
		gO2	134, 203
		gO3	134, 271
		gO4	110, 203
***UL115***	*Eco24I* *CseI*	gL1	40, 90, 100, 108, 133, 150
		gL2	40, 100, 133, 150
		gL3	100, 133, 150, 165, 225
		gL4	90, 100, 133, 165

bp, base pairs.

### CMV UL128 locus genotyping

To successfully amplify the genes encoding the CMV UL128L, nPCR (*UL130* and *UL131A*) and hemi-nested PCR (*UL128*) assays were used. The sequences of all primers used in UL128L genotyping are listed in Table 6. The reaction mixture for *UL128* and *UL131A* gene amplifications consisted of 0.5 μg (blood) or 0.2 μg (urine) template DNA (5 μl) and 45 μl reaction solution, as described above, while for the *UL130* gene, it was as follows: 5 μl template DNA, 10 μl 5 × KAPA HiFi Fidelity Buffer, 1.5 μl 10 mM KAPA dNTP, 0.15 μl gene-specific primers, 1 μl KAPA HiFi polymerase (1 U/μl, KAPA Biosystems, Boston, MA, USA), and 32.2 μl nuclease-free water. The *UL128* and *UL131A* reactions were performed at 95 °C for 5 min, followed by 30 cycles of 95 °C for 1 min, annealing at 55 °C for *UL128* and 57 °C for *UL131A* for 1 min, and 72 °C for 1 min, followed by a final extension at 72 °C for 10 min. The PCR was performed in a Veriti® 96 Well Thermal Cycler (Applied Biosystems). The PCR parameters for *UL130* were as follows: 95 °C for 3 min and 35 cycles each at 98 °C for 20 s, at 59 °C for 15 s, and at 72 °C for 15 s with a final extension at 72 °C for 5 min. Positive and negative controls were included in the PCR assays, as described above. The amplicons were verified by the QIAxcel capillary electrophoresis system (Qiagen) and were sequenced using an Applied Biosystems 3730xl sequencer (Applied Biosystems).

CMV sequences were aligned to the reference sequences deposited in the NCBI using BLAST.

### Statistical analysis

The gene variant distributions and the association between them and the outcome of CMV infection were analyzed using Fisher’s exact test or the chi-square test. A nonparametric Mann–Whitney U-test with correction for continuity and Wilcoxon rank-sum test were performed to calculate the *P*-values for the relationship between the viral load and infection outcome. Pairwise correlations between gene variants were evaluated with Spearman’s rank correlation coefficient (ρ). A logistic regression model was used to evaluate the association between the specific CMV variants and clinical characteristics of patients, adjusting for potential cofounders, e.g., DNAemia level. The data were analyzed using descriptive statistics, including the median, range, and 95% confidence intervals (CIs). Analyses were performed using the SPSS statistical software package for Windows 24.0 (SPSS, Chicago, IL). A *P*-value ≤0.05 was considered as statistically significant.
